# Expandable versus static cages in unilateral biportal endoscopy lumbar interbody fusion (ULIF) for treating degenerative lumbar spondylolisthesis (DLS): comparison of clinical and radiological results

**DOI:** 10.1186/s13018-023-03979-z

**Published:** 2023-07-17

**Authors:** Shuyan Cao, Bingjie Fan, Xin Song, Yi Wang, Wenzhe Yin

**Affiliations:** 1 Department of Orthopaedics, Second Affiliated Hospital of Harbin Medical University, Harbin, Heilongjiang China; 2 Department of Oncology, Affiliated Hospital of Guizhou Medical University, Guiyang, Guizhou China; 3 Department of Orthopaedic, The First Affiliated Hospital of Zhengzhou University, Zhengzhou, Henan China

**Keywords:** Expandable cages, Static cages, DLS, ULIF, Radiological result

## Abstract

**Background:**

In recent years, early rehabilitation after spinal fusion and the recovery of physiological curvature have attracted much attention. Therefore, expandable cages have entered the field of vision of scientists. The goal of the current study was to compare the clinical and radiological results of unilateral portal endoscopic lumbar interbody fusion (ULIF) in the treatment of degenerative lumbar spondylolisthesis (DLS) with expandable versus static cages.

**Methods:**

We retrospectively analysed patients who received ULIF treatment for DLS from May 2019 to February 2021. Patients were categorized by cage type (static vs. expandable), and the main study was the preop and postop clinical and radiological index changes of the patients.

**Results:**

Eighty-four patients were included (38 in the static cages group; 46 in the expandable cages group). There was no difference in the preop results between the two groups. The VAS scores for low back and leg pain and ODI scores in the expandable cages group 7 d postop were significantly superior to those in the static cages group (*P* < 0.05), and the segmental angle and PDH in the expandable cages group postop were significantly higher than those in the static cages group (*P* < 0.05). The fusions at 6 m postop in the expandable cages group were superior to those in the Static Cages group (*P* < 0.05).

**Conclusions:**

The results of this study showed that compared with the stable cage group, the expandable cage group had unique advantages in restoring the physiological curvature of the lumbar spine, increasing the fusion rate, and relieving pain in the early postoperative period. ULIF can be used to treat single-segment, mild lumbar spondylolisthesis patients using expandable cages instead of static cages.

## Introduction

Since the term lumbar spondylolisthesis was first coined by Newman and Stone in 1955, a variety of surgical approaches have evolved over the following decades in the development of surgical techniques, while clinical evidence suggests that decompressive fusion is the most effective treatment [1]. In recent years, unilateral biportal endoscopic lumbar interbody fusion (ULIF) has been widely used for the treatment of lumbar spondylolisthesis. ULIF technology uses the surgical approach and method of minimally invasive transforaminal lumbar interbody fusion (TLIF) under unilateral biportal endoscopic (UBE), which can also be called UBE-TLIF [2, 3]. In ULIF, two working channels are established so that the observation channel and the operation channel are separated from each other and do not hinder each other. This not only combines the advantages of an open surgical field of vision and a large operating range but also avoids the damage of minimally invasive TLIF technology to the muscle-ligament structure due to the use of a tubular retractor and can also achieve direct decompression through unilateral discectomy, facetectomy, and bilateral laminoforaminotomy via a unilateral approach bilateral intervertebral foramen incision and intervertebral fusion under direct vision [4–7].

At present, there are various cages on the market, which can be generally divided into three types: static, coplanar expandable intervertebral fusion cages, and biplane expandable intervertebral fusion cages [8]. The essential difference between the expandable cages and the static cages is the size of the original volume. Unlike static cages, expandable cages maintain the minimum volume before reaching the intervertebral space and expand to the required height after being placed within the intervertebral space with special instruments. Previously, due to the limitation of instruments, the application of expandable cages in ULIF technology was very rare. In 2005, GERSTEIN and Shabat et al. [9] reported for the first time the design of a variable-shape B-Twin intervertebral fusion device, which solved the problem that the intervertebral cage was difficult to place due to the limitation of the operating space in minimally invasive surgery. Since then, expandable cages have been increasingly recognized by spinal surgeons. In this study, the expandable cages supported by Ruizhi, Shanghai, Co., China, and the static cages supported by Weigao, Shandong, Co., China, were used to compare the application of the two cages in ULIF and the prognosis of the patients receiving these two types.

Previous studies on ULIF have mainly focused on the clinical functional recovery of patients postoperatively. At the same time, many recent studies have expounded the influence of the changes in pelvic spine parameters in postoperation, especially the reduction in spondylolisthesis and the changes in segmental angle, which makes people pay attention to expandable cages. However, the use of static and expandable cages in ULIF and their effect on changes in spinal radiologic parameters have not been studied. Therefore, we retrospectively analysed the use of static and expandable cages in ULIF for treating degenerative lumbar spondylolisthesis (DLS) as well as the clinical and radiological parameter changes postoperatively.

## Materials and methods

### Ethical approval

This retrospective study was approved by the Ethics Committee of the First Affiliated Hospital of Zhengzhou University, China. The work described has been carried out by The Code of Ethics of the World Medical Association (Declaration of Helsinki) for experiments involving humans. Approved number: 2022KY0771002. All patients signed informed consent forms for surgery preoperatively.

### Inclusion and exclusion criteria

The inclusion criteria were as follows: (1) recurrent lumbosacral pain with or without intermittent claudication; (2) diagnosis of single-segment Meyerding I or II degree vertebral slippage (L2/3, L3/L4 or L4/L5) on radiology; (3) no significant improvement in symptoms after 3–6 months of regular conservative treatment with a clear diagnosis; and (4) combined cauda equina syndrome. The exclusion criteria were as follows: (1) history of previous lumbar spine surgery; (2) spinal infection and tumour; (3) combined lateral kyphosis deformity; and (4) multiple underlying diseases, in which the patient could not tolerate surgery.

### Patient population

Patients diagnosed with DLS in our hospital from May 2019 to February 2021 were collected according to the inclusion and exclusion criteria. All procedures were performed by the same experienced spine surgeon. The general information of the patients was collected, including sex, age, BMI, course of the disease, surgical segment, slippage grade, follow-up time, and whether they had diabetes.

### Operative technique

**Fig. 1:**
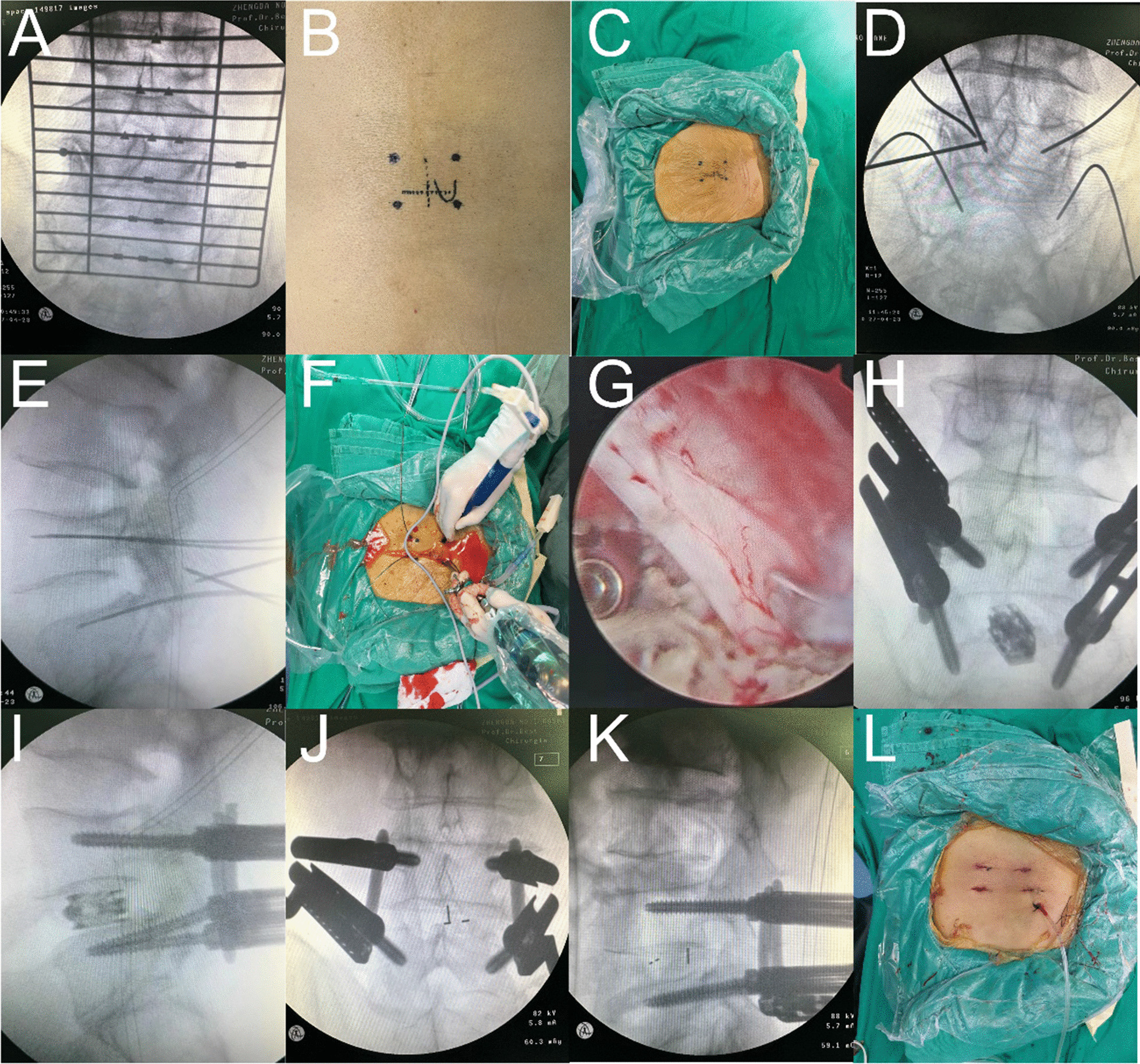
Surgical procedure for ULIF with Static versus Expandable cages

After successful general anaesthesia, the patient was placed in the prone position. Positioning responsible segments and bilateral pedicle surface projections under C-arm fluoroscopy were marked on the skin (Fig. 1A, B). The surgical incision was centred, routine disinfection was performed, and a sterile single fold was placed into a "U" shape, ensuring the smooth flow of lavage fluid out of the surgical area (Fig. 1C). C-arm fluoroscopy was used to place bilateral pedicle percutaneous screw guides for the responsible segment (Fig. 1D, E), and two transverse incisions of approximately 1–2 cm were made at the projection of the superior and inferior pedicles on the side with severe symptoms (Fig. 1F) to establish observation and working channels. After connecting the endoscopic system (KARL STORZ Company, IMAGE1 S camera system), a radiofrequency tool was used for further exposure of the spinous process, lamina, and articular process regions. The dural sac and nerve root were exposed, the nucleus pulposus forceps grasped the protruding nucleus pulposus, the cartilage end plate was scraped, and autologous allogeneic mixed bone particles were implanted.

The Expandable Cages group: A special handle was used to hold the cage (Ruizhi, Shanghai, Co., China) to the intervertebral space and enter the position 3–4 mm away from the posterior edge of the vertebral body (Fig. 1G). Then, the handle was rotated to open the cage to the required height. The area around the nerve root was explored without obvious compression, and the pedicle screw was inserted along the pedicle screw guide pin after exiting the endoscopic system (Fig. 1H, I).

The Static Cages group: Based on the model of the osteotome used for end plate treatment, we roughly predicted the model of the cage (Weigao, Shandong, Co., China) needed and gradually adjusted to the model we needed until the intervertebral height was restored. A pedicle screw was placed along the pedicle screw guide needle (Fig. 1J, K).

The C-arm was subjected to fluoroscopic examination again to confirm that the internal fixation position was good, the incision was cleaned and sutured, and a drainage tube was inserted (Fig. 1L).

### Clinical assessment

The operation time, blood loss, and postoperative hospital stay were recorded. The Oswestry Disability Index (ODI) [10] score and the Visual Analogue Scale (VAS) [11] scores for low back and leg pain were recorded before the operation, 7 d postoperatively, 3 m postoperatively, and at the last follow-up. The clinical efficacy of our patient was assessed at the last follow-up with a modified MacNab [12].

### Radiographic assessment

**Fig. 2:**
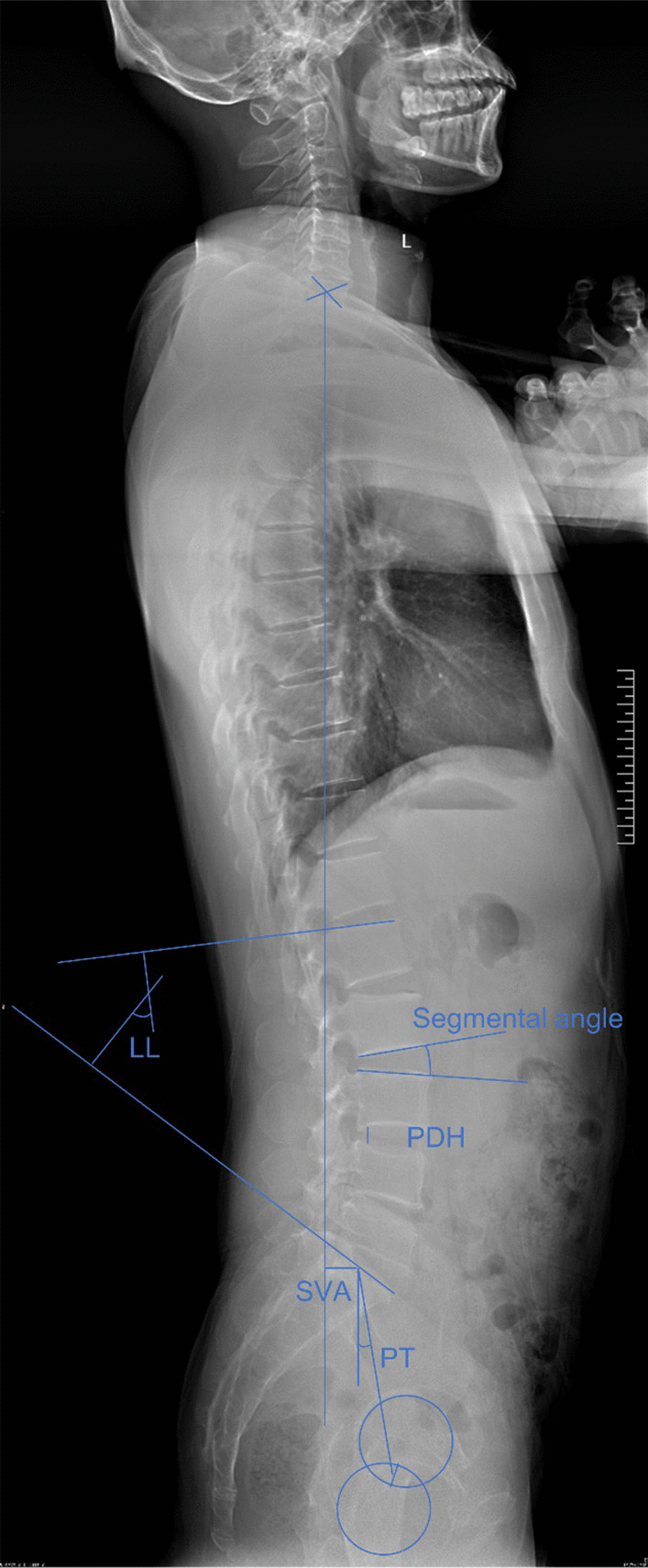
Radiological parameter measurement method

Segmental lordosis (SL), lumbar lordosis (LL), pelvic tilt (PT), sagittal vertical axis (SVA), posterior disc height (PDH), and segmental angle were collected preoperatively and 3 months postoperatively. Previous studies have shown that the height of the intervertebral foramen is always inaccurate due to posture and other reasons, so we used the PDH as a replacement for the measurement of the intervertebral foramen height [13]. See Fig. 2 for the specific measurement method. At 6 m postoperatively, 1 y postoperatively, and at the last follow-up, the anteroposterior and lateral lumbar radiographs and CT were taken, and the fusion rate was determined by Bridwell's fusion grading system [14]. All radiographic measurements were made by 2 independent observers, and the mean of the values was used for analysis. In the event of a discrepancy, a third senior reviewer was consulted.

### Statistical analyses

All statistical analyses were performed using the Statistical Package for the Social Sciences (SPSS) for Windows version 26.0 (IBM SPSS Statistics for Windows, Armonk, NY, USA). Measurement data are expressed as the mean ± standard deviation. For between-group comparisons, normally distributed variables were assessed using an independent sample *t* test. A Wilcoxon signed-rank test was used to compare the change in radiographic parameters from preoperatively to postoperatively. The VAS and ODI scores in each group at different time points were compared using repeated measures analysis of variance. Chi-square analysis was used to compare the count data. Statistical significance was set at *P* < 0.05, and all *P* values were 2-tailed.

## Results

### General information

Eighty-four patients were included in this study: 46 in the Expandable Cages group and 38 in the Static Cages group. The Expandable Cages group had an average age of 56.83 ± 12.15 yr and 60.87% (*n* = 28) females. The Static Cages group had an average age of 56.69 ± 12.39 yr and 60.53% (*n* = 23) females. No significant differences were detected in basic patient information between the two groups (*P* > 0.05) (Table 1).

**Table 1:** Baseline characteristics of patients

	Cage type		*P* value
	Static cage (*n* = 38)	Expandable cage (*n* = 46)	
Sex			0.97
Male	15	18	
Female	23	28	
Mean age ± SD (y)	56.69 ± 12.39	56.83 ± 12.15	0.96
BMI (kg/m^2^)	23.82 ± 2.85	23.71 ± 2.32	0.86
Minimum BMI	19.14	20.31	
Maximum BMI	27.69	28.52	
The course of the disease (y)			0.58
< 2	20	27	
≥ 2	18	19	
Operation level			0.71
L4/L5	25	32	
L5/S1	13	14	
Lumbar spondylolisthesis index			0.52
1	28	31	
2	9	13	
3	1	2	
Follow-up time (m)	27.45 ± 3.47	26.30 ± 4.23	0.19
Diabetes			0.59
Diabetes	8	12	
No diabetes	30	34	

### Clinical outcome measures

The measurement was repeated for all results, and analysis of variance was performed. There was no significant difference in operation time, blood loss, or postoperative hospital stay between the two groups (*P* > 0.05) (Table 2). However, the VAS scores for low back and leg pain and ODI scores of the two Groups 7 d postop, 3 m postop, and at the last follow-up were significantly improved compared with those during preop (*P* < 0.05). At the same time, we found that 7 d postop, the VAS scores for low back and leg pain and ODI scores in the Expandable Cages group were significantly lower than those in the Static Cages group, and the differences were statistically significant (*P* < 0.05) (Table 3). The clinical efficacy of the patient was assessed at the last follow-up with a modified MacNab score. There was no significant difference in the improvement rate between the Expandable Cages group and Static Cages group (*P* = 0.96, Table 3).

**Table 2:** Comparison of the periop parameters between the two groups

	Static cage	Expandable cage	*P* value
Operation time (min)	169.25 ± 28.37	158.39 ± 31.26	0.10
Blood loss (ml)	138.25 ± 25.91	126.17 ± 31.85	0.06
Postop hospital stay (d)	7.34 ± 2.80	7.15 ± 3.20	0.78

**Table 3:** Clinical efficacy evaluation of the patients in the two groups preop and postop

	Cage type		*P* value
	Static cage (*n* = 38)	Expandable cage (*n* = 46)	
VAS of low back pain			
Preop	6.55 ± 1.78	6.49 ± 1.84	0.88
7d postop*	4.91 ± 1.35	3.98 ± 1.74	**0.01**
3 m postop*	2.64 ± 1.25	2.73 ± 1.46	0.77
Last follow-up*	1.73 ± 0.96	1.65 ± 1.02	0.71
VAS of leg pain			
Preop	6.23 ± 1.45	6.41 ± 1.65	0.62
7d postop*	4.35 ± 1.61	3.51 ± 1.28	**0.00**
3 m postop*	2.49 ± 1.13	2.36 ± 1.49	0.68
Last follow-up*	1.43 ± 0.87	1.61 ± 1.13	0.42
ODI (%)			
Preop	64.72 ± 12.63	66.18 ± 13.52	0.61
7d postop*	33.65 ± 7.91	39.42 ± 10.63	**0.01**
3 m postop*	24.91 ± 8.15	27.63 ± 9.21	0.16
Last follow-up*	15.18 ± 7.34	18.21 ± 9.15	0.10
Modified Macnab score			0.96
Excellent	29	35	
Good	7	8	
Medium	1	2	
Poor	1	1	

Significant values are in bold

*P < 0.05 compared with preop

### Radiographic assessment

**Fig. 3:**
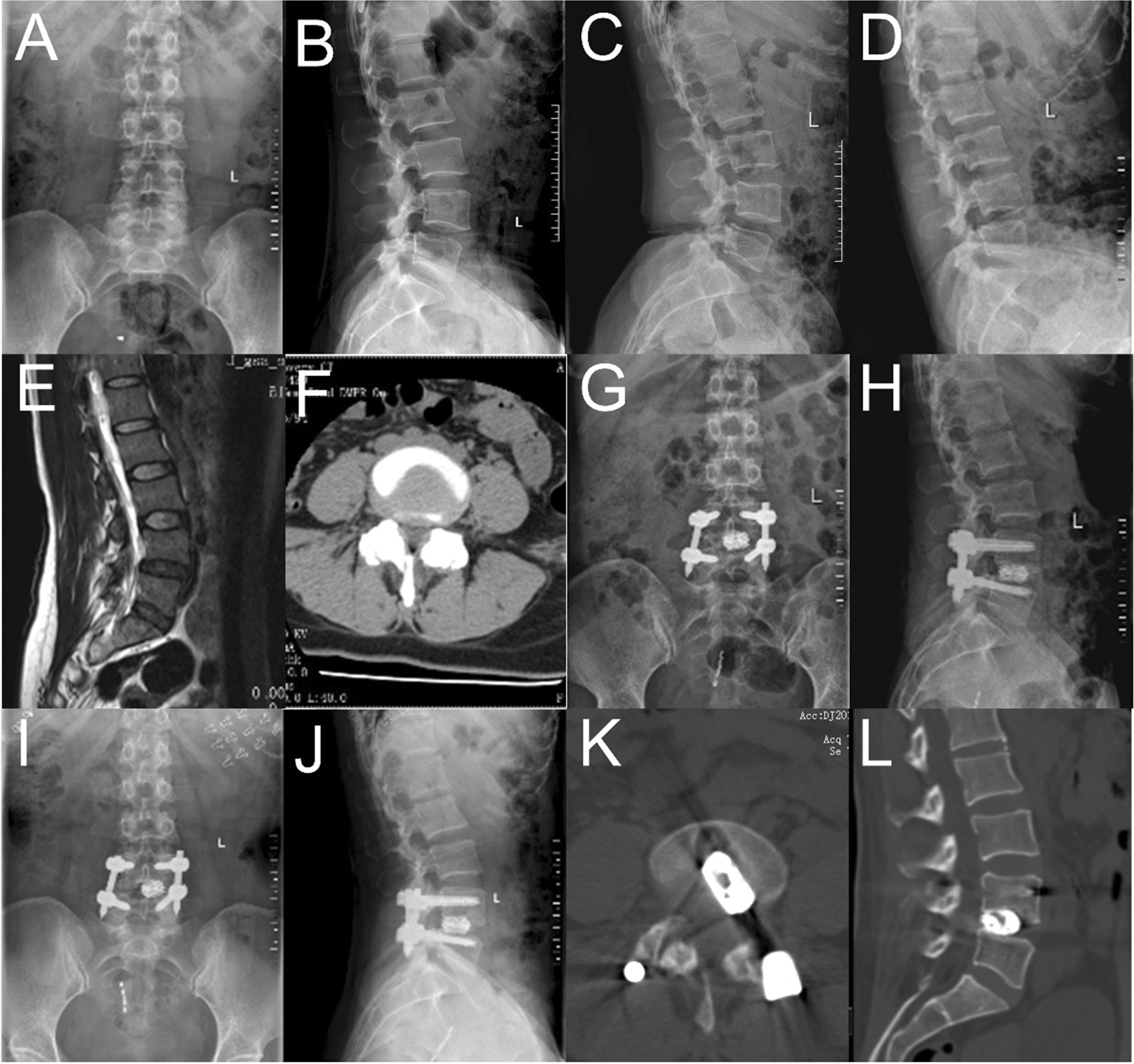
Typical case: **A**–**D** are preoperative anteroposterior, lateral, and dynamic X-ray films of the lumbar spine. **E** is the sagittal plane of preoperative MRI. **F** is the transverse section of preoperative CT. **G** and **H** are anteroposterior and lateral lumbar radiographs at 3 m postop. **I** and **J** are anteroposterior and lateral lumbar radiographs at 1 y postop. **K** and **L** are CT at the last follow-up

The postop spondylolisthesis between the two groups was significantly improved compared with that during preop (*P* < 0.001), but there was no significant difference between the two groups in the postop values (*P* = 0.19). PDH in the two groups postop was improved compared with the preop value. At the same time, we found that the changes in the Expandable Cages group postop were more obvious than those in the Static Cages group (*P* < 0.001). We were surprised to find that in terms of the segmental angle, there was a significant change in the Expandable Cages group postop compared with preop, but no such change was found in the Static cages group. Meanwhile, there was also a significant difference between the Expandable Cages group and the Static Cages group postoperatively (*P* < 0.001). The differences between preoperative and postoperative measurements of LL, SVA, and PT were not significant (Table 4). Bridwell’s fusion grading system was used to estimate lumbar fusion at 6 m postop, 1 y postop, and at the last follow-up. None of the patients underwent reoperation. The difference was statistically significant at 6 m postop; however, there was no significant difference between the two groups at 1 y postop and at the last follow-up (Table 4). We present a typical case of ULIF fusion surgery in Fig. 3. She was a 46-year-old female patient, and she was admitted to the hospital due to "low back pain and numbness of the right lower limb for 2 years, aggravated for 1 month". She was diagnosed with lumbar spondylolisthesis with lumbar instability at L4/L5 and underwent unilateral biportal endoscopic lumbar interbody fusion (Fig. 3).

**Table 4:** Evaluation of radiological indices preoperatively and postoperatively

	Cage type		*P*^#^ value
	Static cage (*n* = 38)	Expandable cage (*n* = 46)	
Segmental angle (°)			
Preop	8.46 ± 1.12	8.14 ± 0.97	0.18
Postop	9.07 ± 1.54	12.36 ± 1.69	**0.00**
* P*^∗^ value	0.06	**0.00**	
LL (°)			
Preop	46.81 ± 11.36	48.24 ± 9.27	0.52
Postop	49.31 ± 10.25	50.24 ± 12.79	0.72
* P*^∗^ value	0.32	0.39	
Spondylolisthesis (mm)			
Preop	6.18 ± 1.57	6.57 ± 1.16	0.20
Postop	2.97 ± 0.86	2.74 ± 0.73	0.19
* P*^∗^ value	**0.00**	**0.00**	
PDH (mm)			
Preop	6.48 ± 0.96	6.84 ± 1.19	0.15
Postop	8.46 ± 1.36	10.32 ± 1.87	**0.00**
* P*^∗^ value	**0.00**	**0.00**	
SVA (mm)			
Preop	3.71 ± 1.05	3.59 ± 0.73	0.54
Postop	3.54 ± 0.89	3.78 ± 1.21	0.31
* P*^∗^ value	0.43	0.39	
PT (°)			
Preop	16.89 ± 3.21	17.97 ± 2.65	0.10
Postop	15.73 ± 2.46	16.86 ± 2.97	0.06
* P*^∗^ value	0.08	0.06	
Fusion at 6 m			0.04
Grade 1	16	29	
Grade II	19	16	
Grade III	3	1	
Fusion at 1y			0.47
Grade 1	29	38	
Grade II	9	8	
Fusion at Last Follow-up			0.83
Grade 1	36	43	
Grade II	2	3	

Significant values are in bold

*P*^#^ value for the difference between Static and Expandable cages

*P*^∗ ^value for change from preop to postop

### Operative complications

All patients completed the surgery without serious complications. One patient in the expandable cages group developed a dural laceration. Artificial dural covering was given during the operation, and antibiotics were used to prevent infection postoperatively. The patient recovered smoothly. One patient in the Static Cages group experienced cage settlement during the postoperative reexamination. However, the patient had no clinical manifestation, so the patient was asked to rest more. Osseous fusion was achieved during the reexamination one-year postop.

## Discussion

Lumbar fusion plays an important role in the treatment of lumbar degenerative diseases. In recent years, with the rapid development of endoscopic spine surgery (ESS), it has become one of the least invasive surgical procedures, providing results comparable to traditional open surgery but with less tissue damage, shorter hospital stays, and a quicker return to normal activities [15–18]. The ULIF technique in this study is one of the most studied biportal endoscopic spine surgery (BESS) operations at present [2]. There is no such research on the types of cages used in ULIF and the impact of different cage types on the clinical and radiological results of patients postoperatively. Therefore, we retrospectively analysed the application of two cages that are currently more commonly used in ULIF.

Analysis of the clinical results of the two groups of patients revealed that the VAS scores for low back and leg pain and the ODI scores of the two groups were significantly improved postoperatively. However, the results of the Expandable Cages Group 7 d postop were significantly superior to those of the Static Cages group. On the one hand, the initial height of the expandable cages we used was only 8 mm, and its minimum implantation height ensured that the traction stimulation to the nerve root during the implantation was small. At the same time, the spinous process, the intervertebral ligament, and part of the articular process were preserved, and damage to the posterior column in the spine was minimized. On the other hand, it is possible that the surgeon needs to constantly test the model during the implantation process of the static cage to obtain the appropriate model, to avoid low back pain and reduction of the fusion rate due to the small, selected model, or upper and lower end plate damage and implantation difficulty due to the large selected model. A meta-analysis conducted by Yang et al. [19] showed that reducing nerve root traction and dural sac injury during surgery had positive significance for patients' early rehabilitation after surgery. However, the long-term curative effect after spinal fusion is closely related to adequate decompression during the operation, and the decompression effect of the two groups is the same, so the long-term follow-up results also show that there is no difference between the two groups [20]. Another surprising finding was that although there was no significant difference in operation time between the two groups, we found that the average operation time of the expandable cages group was slightly lower than that of the static cages group. Our analysis may be due to the following: first, the volume of the Expandable cages before implantation was the smallest, and the principle of using a pinion to drive a bull gear after reaching the intervertebral space enabled the surgeon to easily achieve the required intervertebral height with little force, and the surgeon did not need to repeatedly test the model to obtain the required model like the Static group. Second, it is possible that the expandable cages do not need posterior compression after the intervertebral space is expanded, which shortens the operation time.

When comparing the radiological parameters, we found that the spondylolisthesis and PDH of the two groups were significantly better than those preoperatively. At the same time, the improvement of the PDH in the Expandable cages group was significantly better than that in the Static cages group. In terms of the segmental angle, there were significant improvements postop in the Expandable cages group compared with preop, which was not the case in the Static cages group. This may be because the expandable cages we used provided a 3° anterior lobe, which was more in line with our physiological lumbar lordosis angle and reconstructed the mechanics and anatomy of the anterior and middle columns of the spine. He et al. [21]. retrospectively analysed 107 cases and found that better SL recovery had a positive effect on low back pain after lumbar fusion, which was consistent with the result that the early postop ODI and VAS score of low back pain in the Expandable cages group were improved well. A PUBMED database was retrieved for statistical analysis of previous studies. Hawasli et al. [22] reported 48 cases, and Boktor et al. [23] reported 54 cases of the application of expandable cages in MIS-TLIF. After a 2-year follow-up, they found the same results as ours. However, there are also some different views. Yee et al. [24] reported a retrospective comparative analysis study, and the results showed that there was no significant difference in the segmental angle between the Expandable cages and the Static cages.

In our study, we found 1 patient with a torn dura. Artificial dura was applied to cover the ruptured area during the operation, and antibiotics were used to prevent infection after the operation. The patient also did not feel any obvious discomfort. Other literature has also reported that epidural injury is the most common complication of ULIF, with an incidence of approximately 2%, [25] which is also consistent with our results. When collecting the general data of the two groups, we focused on whether they suffered from diabetes. Although there was no significant difference between the results of the two groups, we found that 1 patient experienced cage settlement, while one patient was old and had a history of diabetes for decades. According to a previous study, old age and internal medicine diseases were both risk factors for intervertebral fusion [26], but these factors were significantly lower than the 6–33% reported in the current literature [27, 28].

This study is also limited in that it was a single-centre retrospective study with a small sample size and a lack of longer-term follow-up to prove the reliability of our results. In addition, most of the patients included in our study were patients with a single segment and mild spondylolisthesis, and the efficacy for patients with multiple segments and severe spondylolisthesis remains to be verified. In summary, a large number of multicentre prospective studies are still needed to compensate for the shortcomings and deficiencies of this study.

## Conclusions

The results of this study showed that compared with the stable cage group, the expandable cage group had unique advantages in restoring the physiological curvature of the lumbar spine, increasing the fusion rate, and relieving pain in the early postoperative period. ULIF can be used to treat single-segment, mild lumbar spondylolisthesis patients using expandable cages instead of static cages.

## Data Availability

The datasets used and/or analysed during the current study are available from the corresponding author on reasonable request.

## Abbreviations

SL: Segmental lordosis
LL: Lumbar lordosis
PT: Pelvic tilt
SVA: Sagittal vertical axis
PDH: Posterior disc height
ULIF: Unilateral portal endoscopic lumbar interbody fusion
VAS: Visual analogue scale
ODI: Oswestry disability index
DLS: Degenerative lumbar spondylolisthesis
UBE: Unilateral biportal endoscopic
TLIF: Transforaminal lumbar interbody fusion
ESS: Endoscopic spine surgery
BESS: Biportal endoscopic spine surgery
